# Comparative study of interhemispheric functional connectivity in left eye monocular blindness versus right eye monocular blindness: a resting-state functional MRI study

**DOI:** 10.18632/oncotarget.24487

**Published:** 2018-02-14

**Authors:** Yi Shao, Jing Bao, Xin Huang, Fu-Qing Zhou, Lei Ye, You-Lan Min, Lin Yang, Zubin Sethi, Qing Yuan, Qiong Zhou

**Affiliations:** ^1^ Department of Ophthalmology, The First Affiliated Hospital of Nanchang University, Nanchang 330006, Jiangxi, China; ^2^ Department of Ophthalmology, The People's Hospital of Hubei Province, Wuhan 430060, Hubei, China; ^3^ Department of Radiology, The First Affiliated Hospital of Nanchang University, Nanchang 330006, Jiangxi, China; ^4^ University of Miami, Miami, Florida 33146, USA

**Keywords:** monocular blindness, voxel-mirrored homotopic connectivity, resting state, functional magnetic resonance imaging

## Abstract

**Objective:**

In the present study, we investigated the brain interhemispheric functional connectivity changes in left eye MB versus right eye MB patients by voxel-mirrored homotopic connectivity (VMHC) methods.

**Methods:**

A total of 31 patients with MB (15 with left eye MB and 16 with right eye MB), and 31 healthy controls (HCs) closely matched for age were recruited. All subjects underwent functional magnetic resonance imaging (fMRI) examinations. The VMHC method was used to evaluate directly functional interactions between the hemispheres. A one-way ANOVA was performed to determine the regions in which the VMHC differs between the three groups. Patients with MB were distinguished from HCs by a receiver operating characteristic (ROC) curve. The relationships between the mean VMHC signal values in many brain regions and clinical features in MB patients were calculated by pearson correlation analysis.

**Results:**

Compared with HCs, MB patients had significantly decreased VMHC values in the cuneus/calcarine/lingual gyrus. Furthermore, left eye MB showed decreased VMHC values in the cuneus/calcarine/lingual gyrus and showed increased VMHC values in the insula and middle frontal gyrus compared with HC. In addition, right eye MB showed decreased VMHC values in the cuneus/calcarine/lingual gyrus, primary motor cortex (M1)/primary somatosensory cortex (S1) and superior parietal lobule.

**Conclusion:**

MB subjects showed abnormal brain interhemispheric functional connectivity in visual pathways. Furthermore, different patterns of brain interhemispheric functional connectivity occurred in the left eye and right eye MB. These VMHC values provide much useful information to explain the neural mechanism changes in MB.

## INTRODUCTION

Blindness is a serious eye disease characterized by loss of response to external light. According to the survey in United States, the incidence of blindness is 1.02 million in 2015 [1]. Blindness can be divided into early blindness and late blindness. Blindness caused by a variety of factors such as cataract [2], glaucoma [3] and ocular trauma [4], *et al.* The surgery and drugs are effective measures to treat reversible blindness. However, there is no effect way to irreversible blindness.

Blind is not only associated with ocular dysfunction, but also with the abnormal visual cortex function. Functional magnetic resonance imaging (fMRI) has been successfully applied to assess the brain function and anatomy changes in blindness. Liu C *et al.* demonstrated that the early blindness showed increased regional homogeneity in the occipital areas [5]. Meanwhile, Liu Y *et al.* exhibited that the early blind showed visual cortex areas [6]. Besides, early blind subjects showed decreased functional connectivity between the primary visual cortex and other Sensory cortex [7]. In addition, the early visual deprivation in blindness is associated morphology of visual cortex changes. Ptito M *et al.* found that congenital blindness showed the atrophy of the visual pathways [8]. Moreover, early blind was associated with decreased gray matter (GM) in the early visual cortex and its linked to the duration of the blindness onset [9]. Dietrich S *et al.* demonstrated that the late blind showed reduced fractional anisotropy (FA) in the optic radiations at either side and the right-hemisphere dorsal thalamus (pulvinar) [10]. Our previous study demonstrated that Late MB subjects were associated with abnormalities of the visual cortex and other vision-related brain regions using regional homogeneity (ReHo) method [11]. The abovementioned studies demonstrated that the blindness showed abnormal brain anatomy and function visual and visual-related regions. However, the understanding of the altered interhemispheric functional synchronization in MB patients remains unknown.

Foubert L *et al.* demonstrated that synchrony of neural activity might be preserved in adult visual cortex despite abnormal postnatal visual experience [12]. Other study reported that interhemispheric synchrony was closely related to the visual experience [13]. Thus, interhemispheric synchrony played a key role in the visual cortex. The VMHC method, a rs-fMRI technology, was used to quantify the functional connectivity(FC) between the time series for a given voxel and that of its mirrored counterpart in the opposite hemisphere [14]. Different from covering baseline brain activity using the amplitude of low frequency fluctuation (ALFF) and regional homogeneity (ReHo) method, the VMHC method was applied to investigate the interhemispheric functional interactions, which reflects the process of exchange and the integration of information between the cerebral hemispheres. The advantage of the VMHC method is that it can reflect the specific patterns of interhemispheric disconnection. The VMHC technology had been successfully applied to investigate the physiological mechanism changes of many disorders including insomnia [15], bipolar disorder [16] and postherpetic neuralgia [17] *et al.*

Here, the aim of our study is to assess the alternations of the interhemispheric functional connectivity in monocular blindness and differences in interhemispheric functional connectivity between left eye MB and right eye MB using the VMHC method.

## RESULTS

### Demographics and visual measurements

The mean values of duration of left eye MB , right eye MB and HC were 25.18 ± 24.15 and 25.29 ± 53.26 and N/A(months). The mean values of best-corrected VA-OD of the left eye MB , right eye MB and HC 0.27 ± 0.50 and 0.85 ± 0.65 and 0.90 ± 0.17. The mean values of best-corrected VA-Os of the left eye MB , right eye MB and HC 0.80 ± 0.26 and 0.25 ± 0.20 and 0.85 ± 0.25 (Table 1).

**Table 1:** Demographics and clinical measurements by group

Condition	Left eye MB	Right eye MB	HC
Male/female	5/10	7/9	20/11
Age (years)	40.91 ± 11.54	43.76 ± 10.37	38.76 ± 10.37
Handedness	15R	16R	31R
Duration (month)	25.18 ± 24.15	25.29 ± 53.26	N/A
Best-corrected VA-OD	0.27 ± 0.50	0.85 ± 0.65	0.90 ± 0.17
Best-corrected VA-OS	0.80 ± 0.26	0.25 ± 0.20	0.85 ± 0.25

Abbreviations: MB, monocular blindness; HC, healthy control; N/A, not applicable; VA, visual acuity.

### VMHC differences

**Figure 1:**
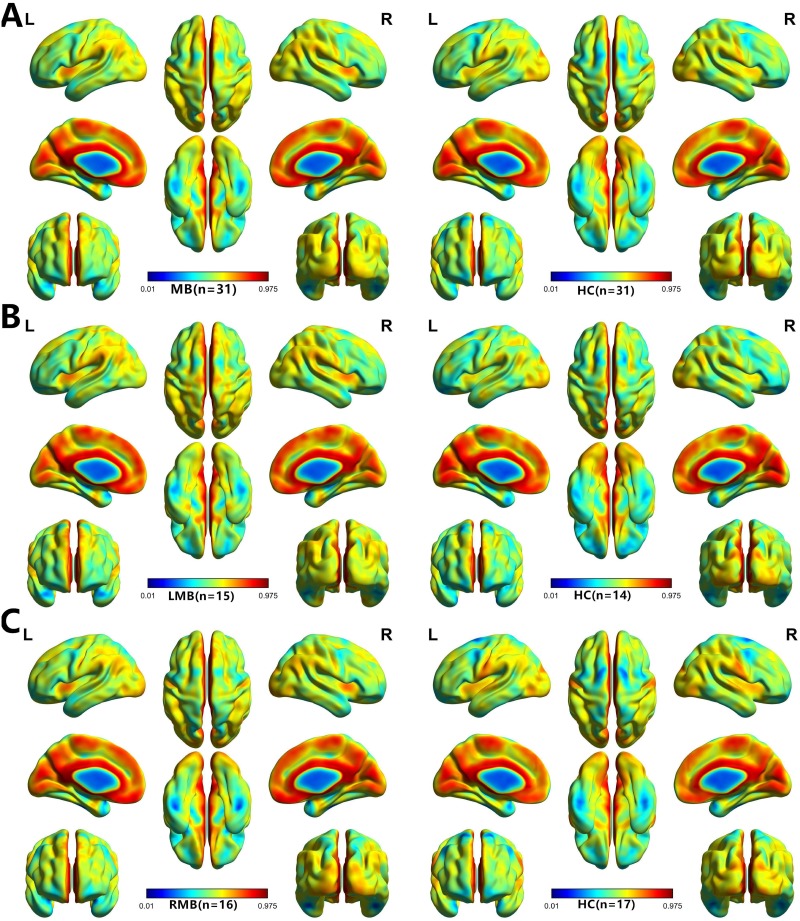
One sample *t*-test results Within-group VMHC maps within the MB (left) and HC (right) (**A**), Left MB (left) and HC (right) (**B**), right MB (left) and HC (right) (**C**) (p < 0.001, FDR corrected). Abbreviations: VMHC, voxel-mirrored homotopic connectivity; MB, monocular blindness; HC, healthy controls; FDR, false discovery rate.

A one-sample *t*-test was performed to extract the VMHC results across the subjects within each group (*P* < 0.05). Intra-group comparison within the MB and left eye MB and right eye MB and HC groups are shown in (Figure 1).

A one-way ANOVA was used to identify regions in which the interhemispheric functional connectivity pattern was different between MB and left eye MB and right eye MB and HCs.

**Figure 2:**
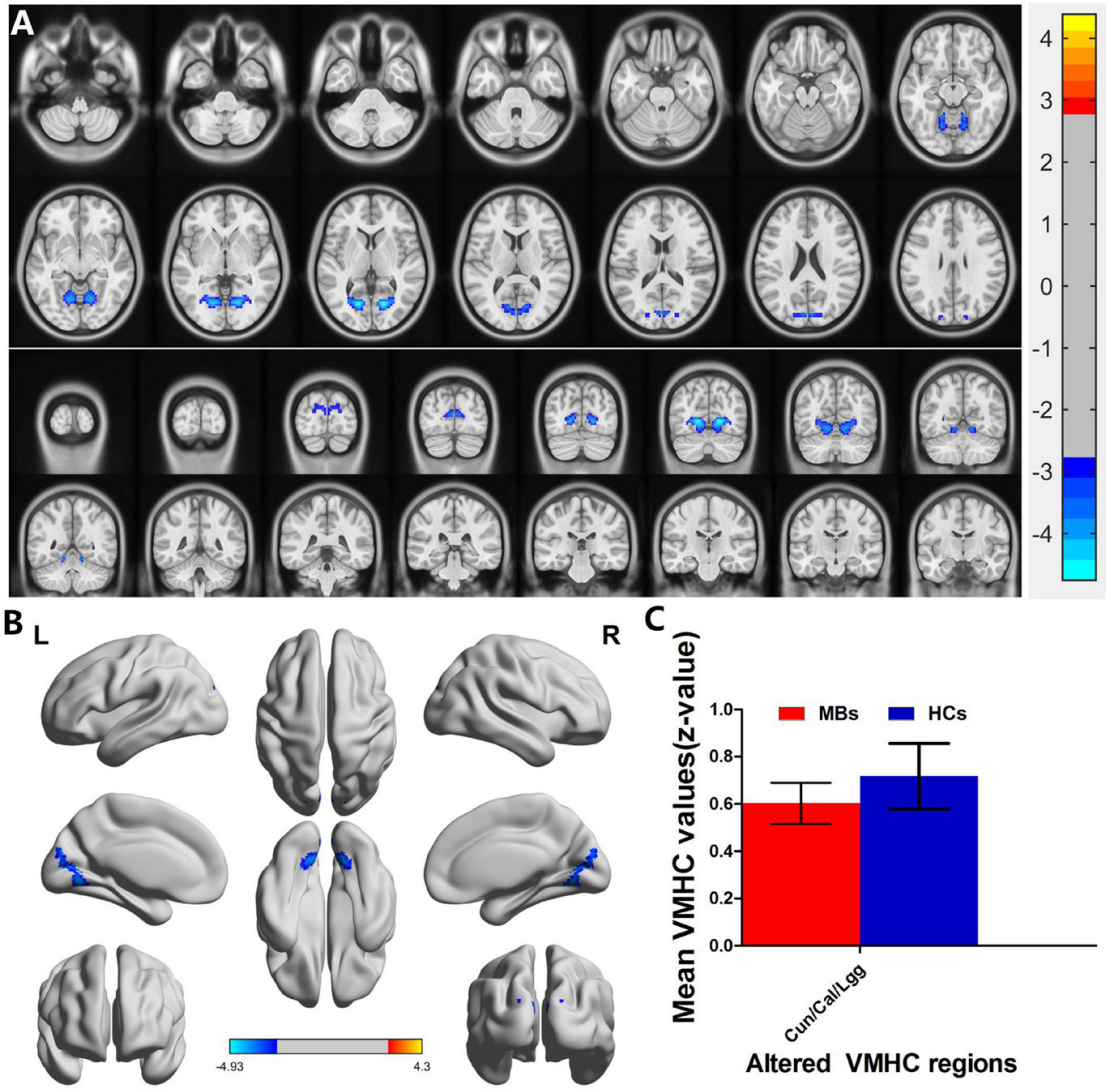
Interhemispheric functional connectivity in the MBs and HCs Significant activity differences were observed in the cuneus/calcarine/lingual gyrus, the blue areas indicate lower VMHC values, respectively (*P* < 0.01 for multiple comparisons using Gaussian Random Field (GRF) theory (*z* > 2.3, *P* < 0.01, cluster > 20 voxels, FDR corrected). (**A**) and (**B**) The mean values of altered VMHC values between the MBs and HCs groups. (**C**) Abbreviations: VMHC, voxel-mirrored homotopic connectivity; MB, monocular blindness; HC, healthy controls; Cun, cuneus; Cal, calcarine; Lgg, lingual gyrus.

MB (*n* = 31) *vs* HC (*n* = 31). Compared with HCs, MB group showed significantly decreased VMHC values in the cuneus/calcarine/lingual gyrus (Figure 2A and 2B [blue] and Table 2). with *P* < 0.01 for multiple comparisons using GRF theory, (*z* > 2.3, *P* < 0.01, cluster >20 voxels, FDR corrected. The mean values of altered VMHC between the two groups are shown with a histogram (Figure 2C).

**Table 2:** Brain areas with significantly different VMHC values between two groups

Conditions	Brain regions	BA	Peak MNI coordinate			Cluster size	*T*- values
			x	y	z		
**MBs (*n* = 31) vs HC (*n* = 31)**							
MB<HC	Cuneus/Calcarine/Lingual Gyrus	17,18,19	±15	−69	0	336	−4.930
**Left eye MBs (*n* = 15) vs HC (*n* = 14)**							
LMB<HC	Cuneus/Calcarine/Lingual Gyrus	17,18,19	±3	−84	18	139	−4.754
LMB>HC	Insula	13	±33	9	9	44	4.388
LMB>HC	Middle Frontal Gyrus	9	±54	21	33	51	4.155
**Right eye MBs (*n* = 16) vs HC (*n* = 17)**							
RMB<HC	Cuneus/Calcarine/Lingual Gyrus	18,19	±30	−66	3	53	−3.785
RMB<HC	M1/S1	3,4,6	±69	−9	15	42	−3.505
RMB<HC	Superior Parietal Lobule	7	±12	−75	57	24	−3.5461

Notes: The statistical threshold was set at the voxel level with *P* < 0.05 for multiple comparisons using Gaussian Random Field (GRF) theory (*z* > 2.3, *P* < 0.01, cluster > 20 voxels, FDR corrected).

Abbreviations: VMHC, voxel-mirrored homotopic connectivity; MB, monocular blindness; HCs, healthy controls; MNI, Montreal Neurological Institute; BA, brodmann area; M1, primary motor cortex; S1, primary somatosensory cortex; L, left; R, right.

**Figure 3:**
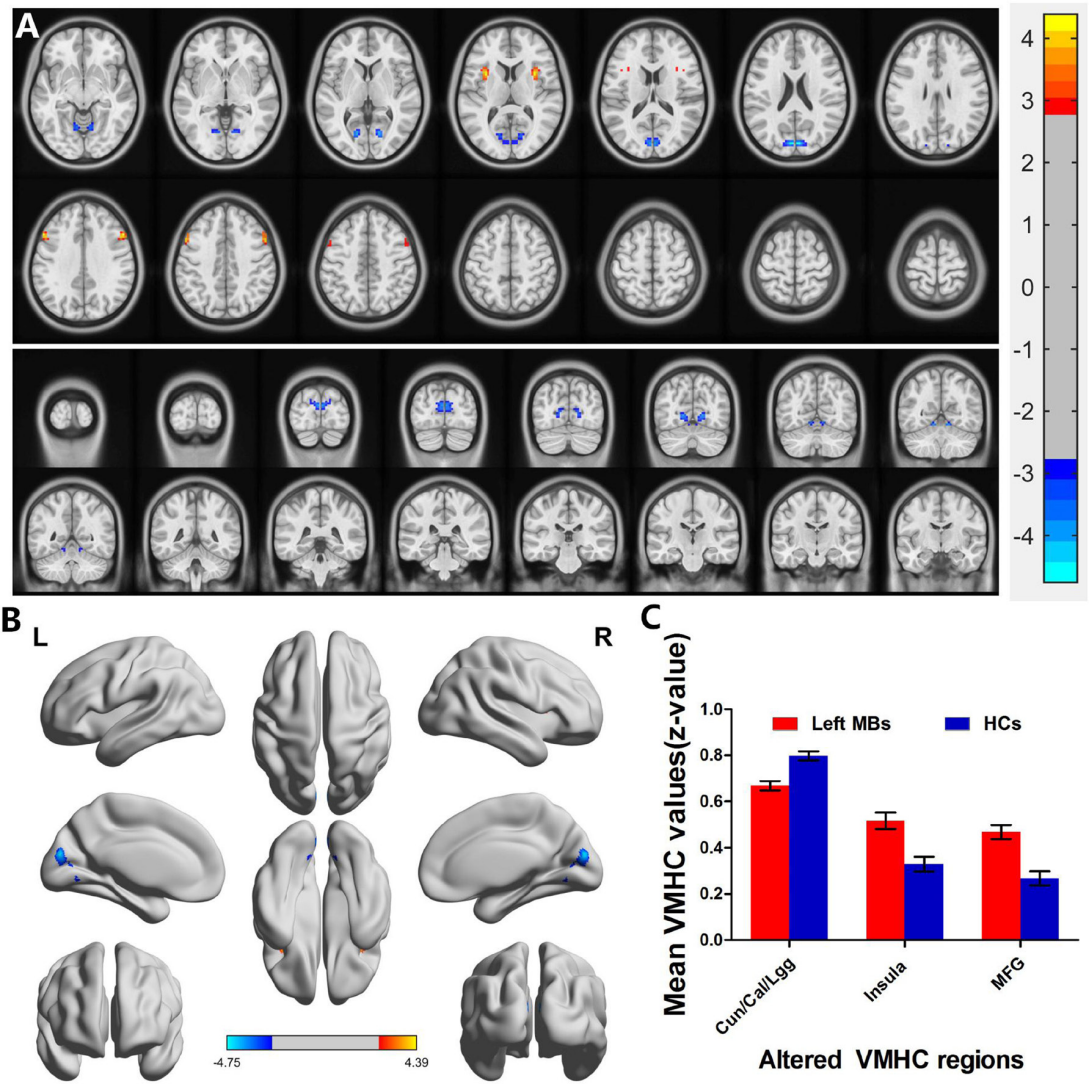
Interhemispheric functional connectivity in the left eye MBs and HCs Significant activity differences were observed in the cuneus/calcarine/lingual gyrus, insula and middle frontal gyrus. The red or yellow denotes higher VMHC values, and the blue areas indicate lower VMHC values, respectively (*P* < 0.01 for multiple comparisons using Gaussian Random Field (GRF) theory (*z* > 2.3, *P* < 0.01, cluster >20 voxels, FDR corrected). (**A**) and (**B**) The mean values of altered VMHC values between the left eye MBs and HCs groups. (**C**) Abbreviations: VMHC, voxel-mirrored homotopic connectivity; MB, monocular blindness; HC, healthy controls; Cun, cuneus; Cal, calcarine; Lgg, lingual gyrus; MFG, middle frontal gyrus.

Left eye MB (*n* = 15) *vs* HC (*n* = 14). Left eye MB showed decreased VMHC values in the cuneus/calcarine/lingual gyrus and showed increased VMHC values in the insula and middle frontal gyrus. (Figure 3A and 3B [blue] or [red] and Table 2); with *P* < 0.01 for multiple comparisons using GRF theory, (*z* > 2.3, *P* < 0.01, cluster > 20 voxels, FDR corrected. The mean values of altered VMHC between the two groups are shown with a histogram (Figure 3C).

**Figure 4:**
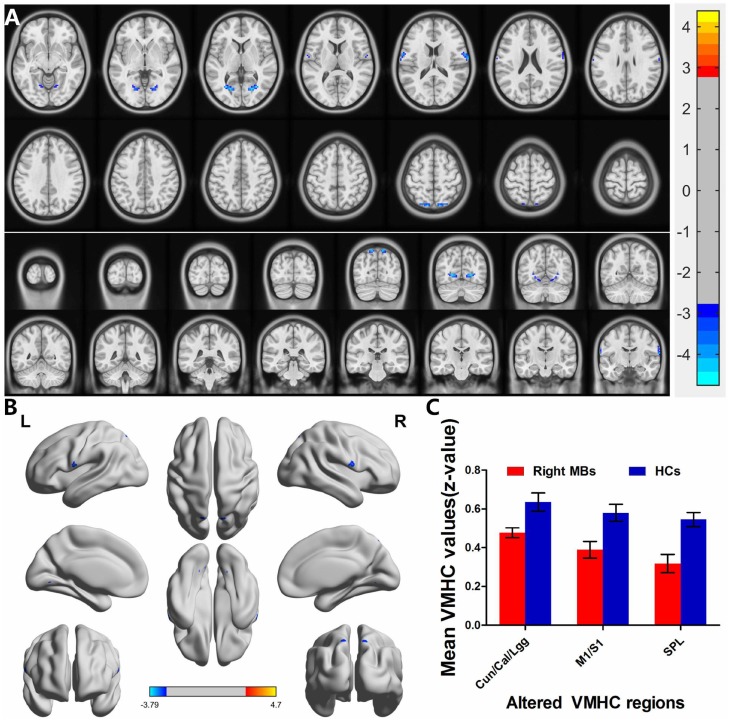
Interhemispheric functional connectivity in the right eye MBs and HCs Significant activity differences were observed in the cuneus/calcarine/lingual gyrus, primary motor cortex (M1)/primary somatosensory cortex (S1) and superior parietal lobule, the blue areas indicate lower VMHC values, respectively (*P* < 0.01 for multiple comparisons using Gaussian Random Field (GRF) theory (*z* > 2.3, *P* < 0.01, cluster >20 voxels, FDR corrected). (**A**) and (**B**) The mean values of altered VMHC values between the right eye MBs and HCs groups. (**C**) Abbreviations: VMHC, voxel-mirrored homotopic connectivity; MB, monocular blindness; HC, healthy controls; Cun, cuneus; Cal, calcarine; Lgg, lingual gyrus; M1, primary motor cortex; S1, primary somatosensory cortex; SPL, superior parietal lobule.

Right eye MB (*n* = 16) *vs* HC (*n* = 17). Compared with HCs, right eye MB showed decreased VMHC values in the cuneus/calcarine/lingual gyrus, primary motor cortex (M1)/primary somatosensory cortex (S1) and superior parietal lobule. (Figure 4A and 4B [blue] and Table 2). with *P* < 0.01 for multiple comparisons using GRF theory, (*z* > 2.3, *P* < 0.01, cluster > 20 voxels, FDR corrected. The mean values of altered VMHC between the two groups are shown with a histogram (Figure 4C).

### Receiver operating characteristic (ROC) curve

**Figure 5:**
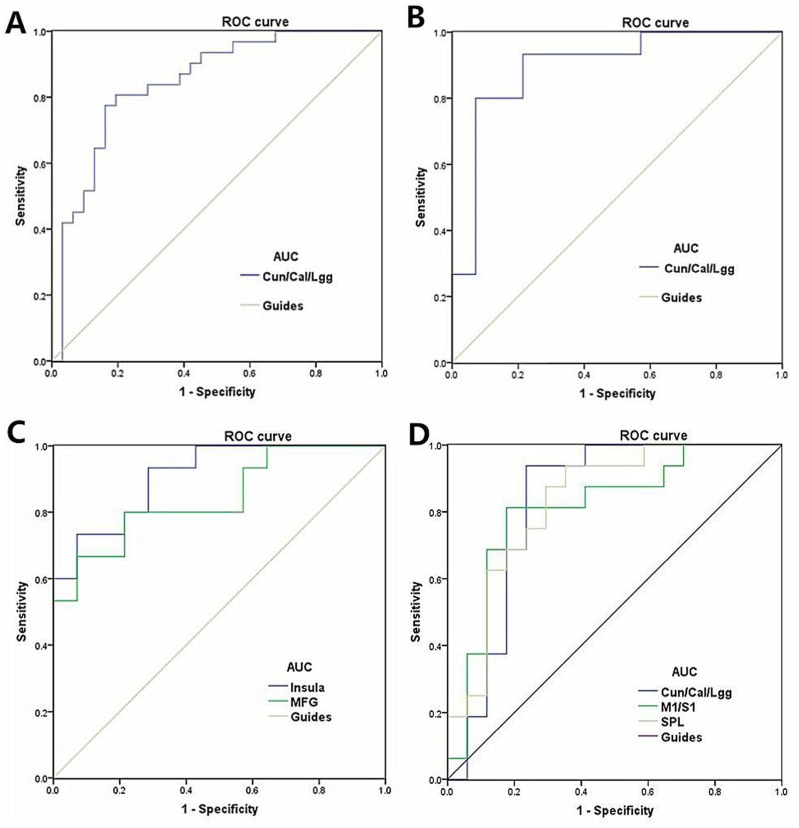
ROC curve analysis of the mean VMHC values for altered brain regions Notes: (**A**) ROC curve: MB < HC, for the Cun/Cal/Lgg 0.845 (*p* < 0.001; 95% CI: 0.746–0.944); (**B**) ROC curve: LMB < HC for the Cun/Cal/Lgg 0.895(*p* < 0.001; 95% CI: 0.772–1.000); (**C**) ROC curve: LMB > HC for the Insula 0.910 (*p* < 0.001; 95% CI: 0.808–1.000) and MFG 0.843 (*p* = 0.002; 95% CI: 0.700–0.986) ; (**D**) ROC curve: RMB < HC for the Cun/Cal/Lgg, 0.827 (*p* = 0.001; 95% CI: 0.671–0.983) and M1/S1 0.813(*p* = 0.002; 95% CI: 0.658–0.967) and SPL 0.831 (*p* = 0.001; 95% CI: 0.688–0.974; Abbreviations: VMHC, voxel-mirrored homotopic connectivity; ROC, receiver operating characteristic; ReHo, regional homogeneity; CI, confidence interval; MB, monocular blindness; HC, healthy control; Cun, cuneus; Cal, calcarine; Lgg, lingual gyrus; M1, primary motor cortex; S1, primary somatosensory cortex; SPL, superior parietal lobule.

We speculated that the VMHC differences between the two groups might be useful diagnostic markers. Thus, the receiver operating characteristic (ROC) curve method was used to assess the mean VMHC values in the different brain regions. The areas under the ROC curve were as follows: 0.845 for the cuneus/calcarine/lingual gyrus (MB<HC, Figure 5A); 0.895 for the cuneus/calcarine/lingual gyrus (LMB < HC, Figure 5B); 0.910 for the insula and 0.843 for the middle frontal gyrus (LMB > HC, Figure 5C); 0.827 for the cuneus/calcarine/lingual gyrus and 0.813 for the M1/S1 and 0.831 for the superior parietal lobule(RMB < HC, Figure 5D);

## DISCUSSION

To our knowledge, VMHC method is a reliable and noninvasive measure to investigate functional interactions between the hemispheres. we demonstrated that both left eye MB and right eye MB showed decreased VMHC values in the cuneus/calcarine/lingual gyrus compared with HC. However, the left eye MB showed increased VMHC values in the insula and middle frontal gyrus compared with HC. The right eye MB showed decreased VMHC values in the primary motor cortex (M1)/primary somatosensory cortex (S1) and superior parietal lobule.

### The analysis of the different VMHC values of MB and HC

The cuneus (BA17), known as visual area 1(V1), is a part of the occipital lobe , which is involved in the visual processing. The primary visual cortex input visual information from the bilateral lateral geniculate body [18]. During natural vision, the V1 processing visual imformation with sparse coding and decorrelation [19]. Besides, the V1 is closely related to visuospatial integration [20]. Moreover, The V1 is the core part of the visual pathways [21]. In our previous study, we demonstrated that the MB showed decreased amplitude of low-frequency fluctuation in right cuneus [22]. Besides, A recent research exhibited that the MB patients showed lower gray matter volume in the bilateral superior lateral occipital cortices compared with health controls [23]. With consistency of these findings, we also found that MB patients had decreased VMHC values in the cuneus, which indicates impairment of the interhemispheric functional connectivity (FC) in the cuneus. Thus, we speculated that the MB might lead to the impaired bilateral coordination dysfunction in V1.

The lingual gyrus, known as BA18, which involves in the visual processing [24]. Besides, the lingual gyrus play an critical role in the spatial memory [25] and reading [26]. The lingual gyrus (BA18) is located in the visual cortex 2 (V2), which is an important area in the visual cortex. V2 is a visual association area, which receives strong feedforward connections from V1. Besides, the V2 play an important role in object shape visual [27] and involves in stereo vision [28]. In our study, we exhibited that MB patients showed decreased VMHC values in the lingual gyrus, which reflect the abnormal interhemispheric functional connectivity (FC) in the lingual gyrus. Thus, we speculated that the MB might lead to the impaired bilateral coordination dysfunction in V2.

### The analysis of the different VMHC values of left eye MB and HC

The critical role of the insular is emotion processing [29]. Besides, the insular is involved in pain and pain-related processing [30, 31]. We demonstrated that the left eye MB showed increased VMHC values in the insula. We speculated that the left eye MB might lead to the dysfunction of emotion.

The middle frontal gyrus (MFG) is part of the frontal gyrus. The MFG plays a critical role in the advanced cognitive function [32]. Besides, the MFG involves in attention [33]. The area under the ROC curve in MFG was 0.843. In our study, we found that the left eye MB exhibited increased VMHC values in the MFG. Our results suggest that the left eye MB might associate with the dysfunction in attention.

### The analysis of the different VMHC values of right eye MB and HC

The primary somatosensory cortex (S1) is located in the postcentral gyrus, which is involved in encoding touch and pain [34, 35]. Besides, S1 are closely related to emotion in pain and touch [36]. Moreover, S1 involved in the vision modulates [37, 38] Qin W *et al.* demonstrated that blindness showed decreased long-range functional connectivity density in the primary somatosensory cortices [39]. Sieben K *et al.* demonstrated that anatomical tracing revealed sparse direct connectivity between primary visual (V1) and somatosensory (S1) cortices [40]. In our study, we found that right eye MB showed decreased VMHC values in the S1, which reflect the abnormal interhemispheric functional connectivity (FC) in the S1. We speculated that the right eye MB might lead to the dysfunction in somatosensory function.

The primary motor cortex (M1), known as BA 4, plays a critical role in motor performance [41, 42]. Besides, the M1 involves in motor learning [43]. The M1 is related to the non-motor cognitive action [44]. In our study, we exhibited that the right eye MB showed decreased VMHC values in the M1, which reflect the impaired interhemispheric functional connectivity (FC) in the M1. Thus, our results suggest that the right eye MB might associate with the dysfunction of motor.

The superior parietal lobule (BA7, SPL) is located between the postcentral sulcus and occipital lobe, which plays an important role in the visuo-motor coordination. The SPL is the part of the visual pathway [45]. Besides, the SPL involves in audio-visual multisensory [46] and language processing [47]. A previous study demonstrated that the early blindness exhibited decreased functional connectivity between the left V1 and the bilateral superior parietal lobule [47]. With support of these findings, we found that the right eye MB showed decreased VMHC values in the SPL, indicating the abnormal interhemispheric functional connectivity (FC) in the SPL. Therefore, we speculated that the right eye MB might lead to the impaired visuo-motor function.

## MATERIALS AND METHODS

### Subjects

In total, A total of 31 patients with MB (15 with left eye MB (5 males and 10 females) and 16 with right eye MB (7 males and 9 females)) were enrolled in the study, all subjects were from the Ophthalmology Department of the First Affiliated Hospital of Nanchang University in Jiangxi province of China. The criteria for MB in the study were: 1) late stage of MB (in 20 MB patients was caused by keratitis; 11MB patients was due to ocular trauma); 2) best-corrected vision of blind eye < 0.05 3) contralateral eye best-corrected vision acuity (VA) ≥ 1 1.0.

The exclusion criteria were: 1) conditions of psychiatric disorders, 2) diabetes, cardiovascular disease, and brain disease (cerebral hemorrhage, cerebral infarction, cerebral vascular malformation).

Thirty-one health controls (20 males and 11 females) with similar age range, were enrolled in the study. All HCs met the following requirements: 1) normal brain parenchyma on cranial MRI; 2) without any ocular disease with visual acuity (VA) ≥ 1; 3) no psychiatric disease (depressive disorder, delusional disorder); and 4) accessible to the MRI scanning (e.g. no cardiac pacemaker or implanted metal devices).

The protocol of the study was approved by the committee of the medical ethics of the Ophthalmology Department of the First Affiliated Hospital of Nanchang University. All of the participants signed the informed consent.

### MRI data acquisition

Imaging was performed on a 3.0 T MRI system with eight-channel head coil (Siemens, Munich, Germany). The whole-brain T1-weighted were obtained with spoiled gradient-recalled echo sequence with the parameters: (repetition time = 1,900 ms, echo time = 2.26 ms, thickness = 1.0 mm, gap = 0.5 mm, acquisition matrix = 256 × 256, field of view = 250 × 250 mm, flip angle = 9°). Functional images with the parameters (repetition time = 2,000 ms, echo time = 30 ms, thickness =4.0 mm, gap = 1.2 mm, acquisition matrix = 64 × 64, flip angle = 90°, field of view = 220 × 220 mm, 29 axial) were corrected.

### fMRI data preprocessing

The Functional data were analyzed using the Data Processing Assistant for Resting-State fMRI Advanced Edition (DPARSFA; http://rfmri.org/DPARSFA) and Statistical Parametric Mapping (SPM8) (http://www.fil.ion.ucl.ac.uk/spm) on the basis of MATLAB2010a (Mathworks, Natick, MA, USA). 1)The first ten volumes of each subject were discarded due to the signal reaching equilibrium and the participants adapted to the scanning noise. The remaining 230 volumes were corrected for delay in acquisition time between different slices and corrected for geometrical displacements according to the estimated head movement. 2) Each subject showed a maximum displacement of less than 1.5 mm in any cardinal direction (x, y, z) and a maximum spin (x, y, z) of less than 1.5°. 3) after motion correction, each T1 image was co-registered to the mean functional image, then were segmented into gray matter, white matter and cerebrospinal fluid;4) the functional images were normalized to the Montreal Neurological Institute space following motion correction and was then re-sampled to a 3×3×3 mm 3 voxel using these parameters estimated during unified segmentation; 5) spatial smoothing of the normalized images were then performed using a 6 mm full width half maximum Gaussian kernel; 6)A multiple regression method was performed to remove possible sources of artifacts, including estimated motion parameters, ventricular and white matter regions, and global signal. 7) A temporal filter (0.01–0.08 Hz) was applied to reduce the effect of low-frequency drift and high-frequency noise. Subsequently, the images of each was used to compute the VMHC.

### VMHC statistical analysis

The individual VMHC maps were converted to *z* values using a Fisher z-transformation to improve the normality with REST software (http://resting-fmri.sourceforge.net). The individual z-maps were entered into a random effect two-sample *t*-test with the global VMHC as covariate in a voxel-wise manner to identify the difference in VMHC between two groups (FDR corrected, *P* < 0.05 and cluster>20). More details were shown in a previous study [48].

### Statistical analysis

The cumulative clinical measurements, including the duration of the onset of MB were analyzed in the study with independent sample *T* test using SPSS version 16.0 (SPSS Inc, Chicago, IL, USA). (*P* < 0.05 significant differences).

A one-way ANOVA was used to identify regions in which the interhemispheric functional connectivity pattern was different between MB and left eye MB and right eye MB and HCs. We performed a post-hoc analysis tests after regressing out age and gender effects to compare the VMHC values between each pair of groups. (*P* < 0.01 for multiple comparisons using Gaussian Random Field (GRF) theory (*z* > 2.3, *P* < 0.01, cluster > 20 voxels, FDR corrected).

The mean VMHC values in the different brain regions between two groups was analyzed by receiver operating characteristic (ROC) curves method. Pearson correlation was used to evaluate the relationship between the mean VMHC values in different brain regions in the MB group and behavioral performances using SPSS version 16.0 (SPSS Inc, Chicago, IL, USA). (*P* < 0.05 significant differences).

## CONCLUSIONS

In summary, MB subjects showed abnormal brain interhemispheric functional connectivity in visual pathways. Furthermore, different patterns of brain interhemispheric functional connectivity occurred in the left eye and right eye MB. These VMHC values could become useful clinical indicators of dysfunctional brain activity in MB patients.

### Limitations

First, the MB enrolled in the study were relatively small sample size, which might have bad affect on the accuracy of the result. Second, the MB subjects were associated with different the duration of the onset of MB, which might have bad influence on the VMHC in the MB. Third, the exact substrate behind the reduced VMHC has not been clear. Future studies may solve this by combining multimodal MRI method, such as diffusion tensor imaging (DTI) and arterial spin labeling (ASL).
